# MIDESP: Mutual Information-Based Detection of Epistatic SNP Pairs for Qualitative and Quantitative Phenotypes

**DOI:** 10.3390/biology10090921

**Published:** 2021-09-16

**Authors:** Felix Heinrich, Faisal Ramzan, Abirami Rajavel, Armin Otto Schmitt, Mehmet Gültas

**Affiliations:** 1 Breeding Informatics Group, Department of Animal Sciences, Georg-August University, Margarethe von Wrangell-Weg 7, 37075 Göttingen, Germany; faisal.ramzan@uni-goettingen.de (F.R.); abirami.rajavel@uni-goettingen.de (A.R.); armin.schmitt@uni-goettingen.de (A.O.S.); 2 Center for Integrated Breeding Research (CiBreed), Albrecht-Thaer-Weg 3, Georg-August University, 37075 Göttingen, Germany; 3 Faculty of Agriculture, South Westphalia University of Applied Sciences, Lübecker Ring 2, 59494 Soest, Germany

**Keywords:** mutual information, epistatic interactions, genome-wide association studies, single-nucleotide polymorphism

## Abstract

**Simple Summary:**

The interactions between SNPs, which are known as epistasis, can strongly influence the phenotype. Their detection is still a challenge, which is made even more difficult through the existence of background associations that can hide correct epistatic interactions. To address the limitations of existing methods, we present in this study our novel method MIDESP for the detection of epistatic SNP pairs. It is the first mutual information-based method that can be applied to both qualitative and quantitative phenotypes and which explicitly accounts for background associations in the dataset.

**Abstract:**

The interactions between SNPs result in a complex interplay with the phenotype, known as epistasis. The knowledge of epistasis is a crucial part of understanding genetic causes of complex traits. However, due to the enormous number of SNP pairs and their complex relationship to the phenotype, identification still remains a challenging problem. Many approaches for the detection of epistasis have been developed using mutual information (MI) as an association measure. However, these methods have mainly been restricted to case–control phenotypes and are therefore of limited applicability for quantitative traits. To overcome this limitation of MI-based methods, here, we present an MI-based novel algorithm, MIDESP, to detect epistasis between SNPs for qualitative as well as quantitative phenotypes. Moreover, by incorporating a dataset-dependent correction technique, we deal with the effect of background associations in a genotypic dataset to separate correct epistatic interaction signals from those of false positive interactions resulting from the effect of single SNP×phenotype associations. To demonstrate the effectiveness of MIDESP, we apply it on two real datasets with qualitative and quantitative phenotypes, respectively. Our results suggest that by eliminating the background associations, MIDESP can identify important genes, which play essential roles for bovine tuberculosis or the egg weight of chickens.

## 1. Introduction

The development of high-density arrays for genotyping in recent years has allowed genome-wide association studies (GWAS) to become powerful tools for the detection of single-nucleotide polymorphisms (SNPs) that are associated with traits of interest. However, GWAS methods are usually based on the analysis of single loci, ignoring the potential interaction between genes, and are therefore of limited applicability for traits that are controlled by multiple genes with possibly complex interactions [1,2,3]. These genes may have only a small effect on the phenotype and could therefore be missed by single-locus analyses despite having a strong influence based on their interactions [4,5,6,7]. While large parts of phenotype variance are attributed to individual SNP effects, these interactions, which are commonly referred to as epistasis, have been shown to be of importance for many complex diseases in humans such as asthma [8], cancer [9] or diabetes [10], as well as for quantitative traits in animals [3,11,12,13,14,15] and plants [16,17,18,19,20], and could help to explain the relationship between the genetic variants and the corresponding phenotype [2,13,21,22].

Due to the large number of possible combinations of SNPs even if only pairwise interactions are considered, the detection of epistasis is a computational challenge, for which a large number of algorithms have been proposed. These methods can be divided into different categories depending on their search strategy. Exhaustive search strategies test every possible combination of SNPs for significance, which often results in a long execution time and can become infeasible for large datasets. This strategy has been used by partitioning methods such as the Combinatorial Partitioning Method (CPM) [23] and the Restricted Partition Method (RPM) [24], as well as several other methods [9,25,26]. Stochastic methods, on the other hand, use random sampling to increase their efficiency, but their results and performance can depend on variables determined by the user. Bayesian Epistasis Association Mapping (BEAM) [27], for instance, applies Markov chain Monte Carlo to compute the posterior probability for association between SNPs and a disease. Its extension epistatic MOdule DEtection (epiMODE) [28] uses Gibbs sampling with a reversible jump Markov chain Monte Carlo to find epistatic interactions. Machine learning methods such as neural networks [29,30,31,32], decision trees [33] or random forest [34,35,36,37] have also been utilized for epistasis detection. Step-wise approaches form a fourth category of algorithms, which first filter out SNPs with a very small or no association signal, and then test among the surviving SNPs for epistatic interactions. BOolean Operation-based Screening and Testing (BOOST) [38], as an example, first performs a likelihood ratio test to filter out unimportant SNPs and then performs an exhaustive search on the others. Leem et al. [39] utilized a k-means clustering of the SNPs and then searched for interactions between SNPs in different clusters. Other methods still use the results of lower-order interactions to find higher-order interactions in an efficient way [40,41].

Several of these methods use information-theory-based measures such as mutual information to quantify epistatic interactions [39,41,42,43,44,45,46]. These measures consider the SNPs and phenotypes as random variables, which allows them to quantify the amount of information, or uncertainty, that is inherent to an SNP or a phenotype and to compute how much information is shared between them, and thereby the strength of association [42]. This approach is model-free and therefore has the advantage of not requiring any prior assumptions regarding the structure of the interactions. By considering all genotype combinations of the SNPs as separate categories, this strategy also avoids the problem of choosing an appropriate encoding method for the SNPs and their interactions, which has been shown to influence the result of regression-based methods [47,48,49]. Nevertheless, the application of information-theory-based approaches has so far been limited to case–control phenotypes. This is because, while the mutual information between two discrete variables can be efficiently calculated using simple contingency tables, the mutual information between a discrete and a continuous variable requires computationally more challenging approaches for an accurate estimation.

Furthermore, the methods mentioned above do not take into account different types of obstacles resulting from sample structure, relatedness between the genotyped individuals or marginal effects of single SNPs on the phenotype [19,50,51]. Such types of obstacles can lead to background associations between SNP pairs and the phenotype, and thus the importance of some SNPs in the epistatic interactions could be overestimated. Consequently, the prediction of most existing methods could be biased, potentially impeding the identification of correct epistatic signals. Hence, elimination of the bias inherent in the genotype–phenotype datasets is needed to separate the signal caused by functional interactions from the background associations between SNPs [19,50].

In this paper, we propose a novel method called Mutual Information-based Detection of Epistatic SNP Pairs (MIDESP) for the detection of pairwise epistatic interactions, which extends the previously mentioned mutual information-based approaches by additionally enabling the identification of epistatic interactions between SNP pairs and quantitative phenotypes. For this purpose we adopt, in the context of epistasis for the first time, the mutual information estimator developed by Ross [52], which accurately estimates the level of epistasis using a *k*th-nearest neighbor-based approach. Moreover, to deal with the possible obstacles inside a genotype–phenotype dataset (as mentioned above), our method incorporates an additional step using the average product correction (APC) theorem [53] to estimate the expected level of background association for each SNP pair. Finally, the removal of the estimated background from the measured epistasis values leads to the detection of correct epistatic signals arising from functional interactions.

In order to demonstrate the performance and functionality of MIDESP, we applied it on two different types of genotype–phenotype datasets. The first type contains several hundred simulated datasets, which we analyzed to optimize the parameters used in the mutual information estimator. On the other hand, the second type contains two further datasets with a qualitative and a quantitative phenotype, respectively. While the dataset with the qualitative phenotype is related to bovine tuberculosis, the other one contains the egg weight of chicken eggs. Our findings show that we are able to successfully reduce the influence of background associations in the prediction of epistatic interactions, which leads to the identification of novel markers/genes that are important to the phenotype of interest.

## 2. Materials and Methods

### 2.1. Data

We analyzed two different datasets, one of which had a qualitative (discrete) case–control phenotype, and the other one had a quantitative (continuous) phenotype. To ensure the data quality, we applied several filters to the datasets following Ramzan et al. [54,55]. We removed SNPs with a minor allele frequency ≤0.01, a genotyping call rate ≤0.97, as well as SNPs significantly deviating from the Hardy–Weinberg equilibrium (*p*–value $<1\times 10^{-6}$). A sample was removed if a phenotype was unavailable for it or if more than 5% of SNPs were missing. Further, we performed linkage disequilibrium (LD) pruning to obtain epistasis results without confounding them through LD [56]. Using PLINK [57], we removed all redundant SNPs with an LD ≥0.99, and thus carrying very similar information about the phenotype. Table 1 gives a short overview of the datasets and their respective sizes.

In the following section, we briefly describe the datasets. Researchers interested in more details about the bovine tuberculosis data are referred to [58] and about the egg weight data to [59].

#### 2.1.1. Bovine Tuberculosis (BT)

This dataset was published by Bermingham et al. [58] and consists of 617,885 SNPs for 1151 cattle. The estimated SNP-based heritability attributable to additive effects for this phenotype is 21% [58]. The cattle belonged to the Holstein–Friesian breed and were collected in Northern Ireland. Genotyping was performed using the BovineHD Genotyping BeadChip. The supplied phenotype is qualitative (case–control) and represents the resistance of the animals towards bovine tuberculosis with 592 cases and 559 controls. Bermingham et al. performed a GWAS on the data to find SNPs associated with resistance to bovine tuberculosis. Overall, they found eight significantly associated SNPs representing two different loci in the genome. After applying our filters 616,398 SNPs remained.

#### 2.1.2. Egg Weight (EW)

The dataset relates to the egg weight (EW) in 36-week-old chickens belonging to a line of Rhode Island Red chicken [59]. While the dataset contains the egg weights for multiple different ages of the chickens, we decided to only use the data for 36-week-old chickens, since this phenotype contains the strongest signal found in previous GWAS [54,59]. For this trait, the estimated SNP-based heritability is 36% [59]. A total of 1063 birds were genotyped using the Affymetrix Axiom^®^ 600 K Chicken Genotyping Array, resulting in an initial set of 580,961 SNPs, which were then filtered. The dataset which was provided by the authors only consists of the 294,705 SNPs that passed their quality filters. We could not remove any further SNPs using our filters.

### 2.2. Method

Based on the number of samples, N, and the number of SNPs, P, we consider a genotype × phenotype dataset as a matrix, $M_{N\times (P+1)}$, where the rows refer to the samples and the columns refer to the phenotype and the SNPs. Furthermore, the phenotype of interest is denoted by $Y^{D}$ and $Y^{C}$ for qualitative (discrete) and quantitative (continuous) traits. Let $S^{i}$ be a sample, let $X^{j}$ be the genotype of an SNP and let $Y^{i}$ be the corresponding phenotype of $S^{i}$. The entry of *M* at position (i,j) is depicted by $X_{j}^{i}$. In the following, we also use *X* and *Y* as placeholders for any of the SNPs or phenotypes, respectively.

**Figure 1:**
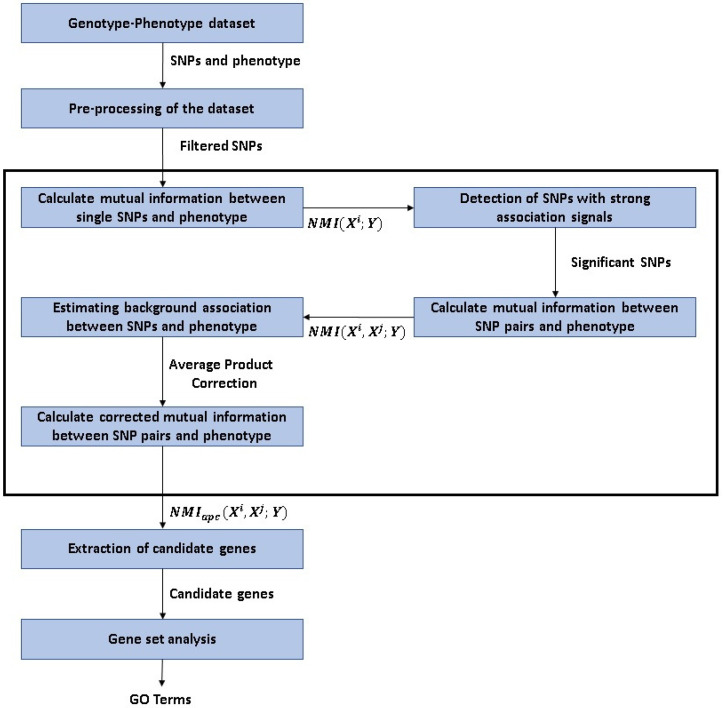
Flowchart of the analysis applied in this study.

An overview of the MIDESP pipeline is shown in Figure 1.

#### 2.2.1. Background on Information Theoretic Measures

In information theory, the entropy, $H\left(X\right)=-\sum_{x\in X}p\left(x\right)\log p(x)$, is a measure for the uncertainty of a discrete random variable, *X*, with alphabet X, which depends solely on its probability function, p(x)=Pr{X=x}, x∈X. It can be interpreted as the amount of information that is necessary to describe the variable on average. By considering the joint probability function, p(x,y), of two discrete random variables *X* and *Y* with alphabets X and Y, this concept can be extended to the joint entropy, H(X,Y), of a pair of variables. Based on these entropies, the mutual information between *X* and *Y* is defined as

(1) MI(X;Y)=H(X)+H(Y)−H(X,Y),

which gives the amount of information that is shared between the variables [60]. The mutual information can be seen as a measure for the association between two variables, which includes linear as well as non-linear dependencies [61].

However, Equation (1) is not applicable if one of the variables is continuous instead of discrete. For a discrete variable *X* and a continuous variable *Y* the MI(X;Y) can be estimated using the *k*th-nearest neighbor-based method developed by Ross [52], which has been shown to be more robust than the commonly used binning-based approaches. The mutual information is estimated as

(2) $$MI\left(X;Y\right)=\frac{1}{N}\cdot \sum_{i=1}^{N}\left(\psi \left(N\right)-\psi \left(N_{x_{i}}\right)+\psi \left(k\right)-\psi \left(m_{i}\right)\right),$$

where:

- ψ(·) is the digamma function;
- $N_{x_{i}}$ for a given sample, $S^{i}$, refers to the number of samples for which the genotype *x* is the same as the genotype $x_{i}$ of $S^{i}$;
- *d* is the distance between sample $S^{i}$ and its *k*th-nearest neighbor $S^{i_{k}}$ with the same genotype as $S^{i}$, defined as the absolute difference between their phenotypes $Y^{i}$ and $Y^{i_{k}}$;
- $m_{i}$ is assigned the number of samples where the absolute difference between their phenotypes and the phenotype $Y^{i}$ is less than or equal to *d*, irrespective of the genotypes.

**Figure 2:**
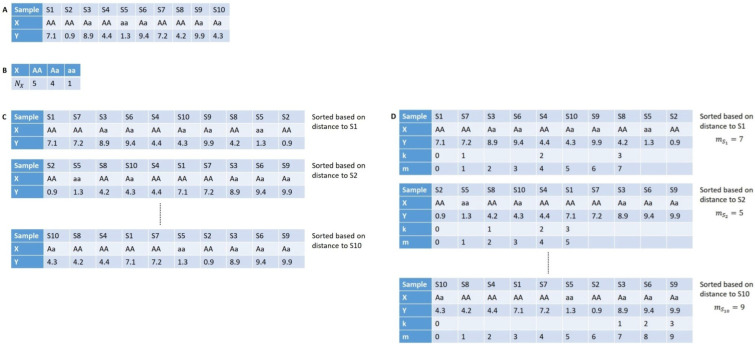
Procedures for estimating the mutual information between a discrete variable *X* as an SNP and a continuous variable *Y* representing a quantitative phenotype using k=3: (**A**) Genotype and phenotype values are given for ten samples S1, S2, ... to S10. (**B**) $N_{x}$ is defined as the number of samples where the genotype is equal to *x*. (**C**) For each sample, $S_{i}$, a sorted list of the samples is created based on the absolute difference between $Y_{i}$ and $Y_{j}$ for sample $S_{j}$. (**D**) The *k*th-nearest neighbor is determined for each sorted list by going along the list and counting the samples that have the same *X* value as the start sample. $m_{S_{i}}$ can then be defined as the index of the *k*th-nearest neighbor in the sorted list. For sample S1 which has the *X* value AA, the sample with the third-closest *Y* value and the same *X* value is sample S8, which has the index 7 in the sorted list. Therefore, $m_{S_{1}}=7$. Based on the $N_{x}$ and $m_{S_{i}}$ values, the mutual information can be estimated.

The identification of these values is shown for a small exemplary dataset in Figure 2.

As shown in Figure 2, only the phenotype *Y* is a continuous variable, hence in general, we can reuse the sorted tables for every SNP by only changing the values of *X*. This allows for an efficient calculation of $m_{i}$. Since MI is only estimated, the resulting values can be outside the range of the valid interval, i.e., [0,H(X)]. Thus, the estimated values outside of this range are set to the closest interval boundary.

#### 2.2.2. Identification of Epistatic Interactions between SNP Pairs

In previous studies [39,42,46], the epistatic interaction between an SNP pair, $X^{i}$ and $X^{j}$, and a qualitative phenotype, *Y*, has been successfully identified by employing the MI metric for which Equation (1) is extended based on the joint entropy $H(X^{i},X^{j})$ as:

(3) $$MI\left(X^{i},X^{j};Y\right)=H\left(X^{i},X^{j}\right)+H\left(Y\right)-H\left(X^{i},X^{j},Y\right),$$

where $H(X^{i},X^{j},Y)$ is the joint entropy of the SNPs $X^{i}$ and $X^{j}$ as well as the phenotype *Y*. However, the concept of MI has not yet been applied to measure the epistatic interaction between an SNP pair and a quantitative phenotype. To the best of our knowledge, we apply for the first time the MI metric for this aim using the following equation:

(4) $$MI\left(X^{i},X^{j};Y\right)=\frac{1}{N}\cdot \sum_{l=1}^{N}\left(\psi \left(N\right)-\psi \left(N_{x_{l}^{ij}}\right)+\psi \left(k\right)-\psi \left(m_{l}\right)\right)$$

In Equation (4), $x_{l}^{ij}$ refers to the joint genotype of the SNP pair $X^{i}$ and $X^{j}$ of sample $S^{l}$.

As shown in [53,62,63], the value of the mutual information and its possible range is strongly dependent on the alphabet size and the marginal distributions of the variables. A normalization of the values is therefore required to address this influence and to make them comparable with each other for further analysis. We apply the following normalization technique based on the entropy and the maximal possible alphabet size of the SNP and SNP pair. Consequently, the MI(X;Y)—and $MI(X^{i},X^{j};Y)$—values are normalized as

(5) $$NMI\left(\ldots ;Y\right)=2\cdot \frac{MI(\ldots ;Y)}{\log\left(\max\left|X\right|\right)+H(\ldots )}$$

#### 2.2.3. Detection of SNPs with Strong Association Signals

As it can be easily seen, the calculation of the pairwise interactions between all SNP pairs requires a quadratic runtime. Therefore, the separation of SNPs with strong association signals from the remaining ones is necessary to reduce the number of pairs under study.

For this purpose, Gültas et al. [63,64] showed that by extending the standard multiple testing theory [65,66], the NMI values can be modeled based on three different distributions: (i) a β distribution $F_{0}$ (null distribution) representing the background signals; (ii) a $G_{1}$ distribution referring to the unrelated associations (in our case between SNPs and phenotype); (iii) a $G_{2}$ distribution modeling the strong association signals (in our case between SNPs and phenotype).

**Figure 3:**
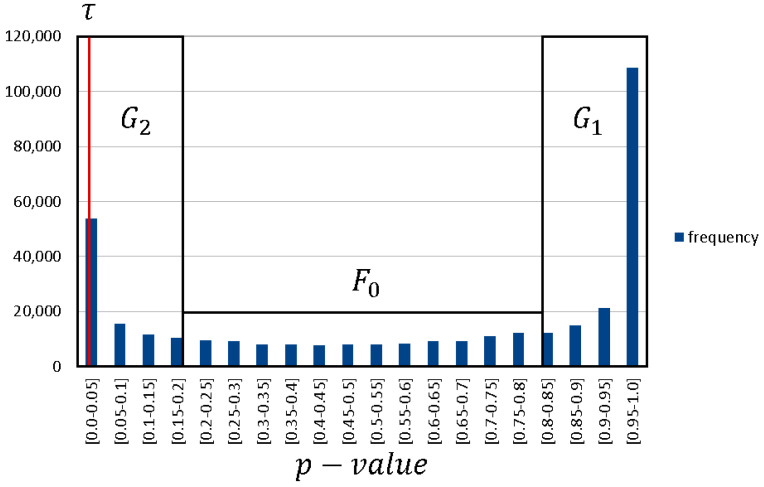
Distribution of *p*–values: the distribution can be divided in three parts, with $G_{2}$ representing the strongly associated SNPs, $G_{1}$ the unrelated SNPs and $F_{0}$ the background. SNPs with a *p*–value less or equal to τ are deemed as significant.

From this follows that $1-F_{0}\left(NMI_{X}\right)$ is the corresponding *p*–value for the association of a SNP *X* to the phenotype. The *p*–value is uniformly distributed over [0,1] if $NMI_{X}$ is $F_{0}$-distributed. However, if *X* belongs to the $G_{1}$ distribution of unrelated SNPs, its corresponding *p*–value is skewed towards 1. On the other hand, if *X* is $G_{2}$ distributed, its *p*–value is skewed towards 0 (see Figure 3).

As the next step, based on two tuning parameters, $\lambda_{1}$ and $\lambda_{2}$, the fraction γ of the $NMI_{X}$ which belong to the background is estimated using Equation (6):

(6) $$\hat{\gamma }=\frac{\mathrm{number}\;\mathrm{of}\;p-\mathrm{values}\;\mathrm{in}\;[\lambda_{1},\lambda_{2}]}{P\cdot (\lambda_{2}-\lambda_{1})}$$

so that the fraction of non-uniformly distributed *p*–values that fall into $\left[\lambda_{1},\lambda_{2}\right]$ is negligible [65,67]. These two parameters are dataset-dependent and are automatically tuned through a trial and error heuristic approach during the analysis [68].

Finally, an SNP *X* is deemed as significant if its *p*–value is less or equal to τ, where τ is a threshold depending on a user-defined false discovery rate, FDR, estimated using Equation (7).

(7) $$\hat{FDR}\left(\tau \right)=\frac{\hat{\gamma }\cdot P\cdot \tau }{\mathrm{number}\;\mathrm{of}\;p-\mathrm{values}\;\leq \tau }$$

For the detection of epistatic interactions using the $NMI(X^{i},X^{j};Y)$ metric, for our further analysis, we only consider SNP pairs where at least one SNP is significant, which results in a reduction in the runtime.

#### 2.2.4. Reduction of the Background Associations between SNPs and Phenotype

As shown in previous studies [19,50,51], a dataset-dependent background association exists between the SNPs and the phenotype that may arise due to population stratification or relatedness of the individuals under study. Such obstacles could interfere with the identification of the correct epistatic signals, and thus could lead to the detection of false positive association signals. Another background association could occur in the detection of epistatic interactions using the NMI metric due to the high level of mutual information between a single SNP and the phenotype. We introduce this issue by way of an example in Section 3.2.

To eliminate these issues to some extent, in our study, we applied the average product correction (APC) introduced by Dunn et al. [53]. The APC theorem is a very successful information-theory-based approach to estimate the expected level of background association between the variables in a dataset. Meckbach et al. [69] showed that this approach is universally applicable, and thus we adopted it for our method. Following this approach, we estimated the expected level of the background between the SNP pair and the phenotype in the calculation of $NMI(X^{i},X^{j};Y)$ as

(8) $$APC\left(X^{i},X^{j};Y\right)=\left(\frac{\bar{NMI_{X^{i}}}\cdot \bar{NMI_{X^{j}}}}{\bar{NMI_{\bar{SNP}}}}\right)$$

In Equation (8), $\bar{NMI_{X^{i}}}$ and $\bar{NMI_{X^{j}}}$ are the average association levels of SNPs $X^{i}$ and $X^{j}$, respectively, in the epistatic interaction:

(9) $$\bar{NMI_{X^{i}}}=\frac{1}{h}\cdot \sum_{l=1}^{h}NMI\left(X^{i},X^{l};Y\right),$$

where *h* is a sufficiently large number (e.g., h>1000) and the SNPs $X^{l}$ are randomly chosen. Further, $\bar{NMI_{\bar{SNP}}}$ denotes the overall average normalized mutual information calculated using a sufficiently large number of NMI values.

Finally, we subtracted the $APC(X^{i},X^{j};Y)$ value of an SNP pair and the phenotype from their initial $NMI(X^{i},X^{j};Y)$ to obtain the corrected $NMI_{apc}\left(X^{i},X^{j};Y\right)$.

#### 2.2.5. Validation of the Epistatic Interactions

To identify the genes pertaining to epistatic SNP pairs, in our analysis, we only considered the *p*-th percentile of the pairs with an $NMI_{apc}$ value > 0. For the interpretation of the interactions, we mapped the SNPs to their corresponding genes based on the mappings provided by the Ensembl database (release 103) [70]. The data were then read into R and a gene–gene interaction network was created with the genes as nodes and their interactions as edges using the igraph package [71]. The number of interactions of a node was termed its degree. In the final step, these degrees were transformed into z-scores and we consequently defined a gene as MIDESP-significant if its z-score was ≥3, as suggested in [69].

To elucidate the biological functions of these genes, we followed previous studies [55,72] and utilized the geneXplain platform [73] to perform a gene set analysis based on the molecular functions of the genes. The results were then visualized in the form of a treemap.

#### 2.2.6. Implementation

The MIDESP pipeline was implemented in Java and is available as a JAR file from https://github.com/FelixHeinrich/MIDESP (accessed on 14 September 2021), allowing for easy usage. The calculations were completely parallelized, allowing for an efficient detection of significant epistatic interactions with a multi-core CPU. Genotype and phenotype information in the form of tped and tfam files were required as input.

## 3. Results

In this paper, we introduce a novel information-theory-based method, MIDESP, for the detection of epistatic interactions using genotype–phenotype datasets. MIDESP is able to analyze both qualitative as well as quantitative phenotypes, unlike previous information theoretical methods [39,41,42,43,44,45,46], which are only applicable to datasets with qualitative phenotypes. Furthermore, our method takes into account the effect of dataset-dependent background associations and eliminates them to some extent using the average product correction (APC) technique [53] to separate correct/functional epistatic signals from those of false positives.

This section consists of four major parts. First, in order to gain insights into the influence of the prerequisite parameter *k* used in Equation (4), we systematically analyzed several simulated datasets to find the most convenient value for it. Second, we introduced, by way of an example, the possible background association effects in epistatic interactions to highlight the importance of the APC approach in our method. In the following sections, we analyzed, by applying MIDESP with a false discovery rate (FDR) of 0.05, two different datasets with qualitative and quantitative phenotypes to demonstrate its functionality.

### 3.1. Analysis of Simulated Datasets for Parameter Setting

**Figure 4:**
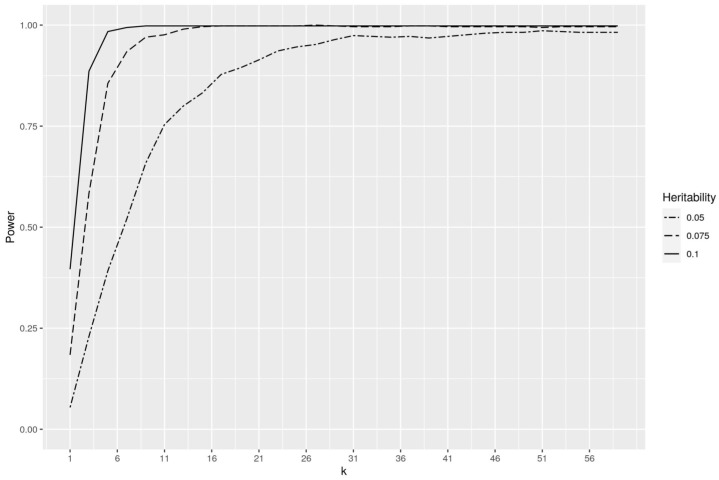
Analysis of simulated datasets for parameter setting of *k*.

Today, it is well established that mutual information is an appropriate metric to measure the association between SNPs and qualitative (case–control) phenotypes [39,44,46,74,75,76,77]. However, we apply here for the first time this metric to quantitative traits. Therefore, we analyzed several simulated datasets to identify a proper value of *k*, which is necessary for the MI estimator (see Equations (2) and (4)). For this purpose, we employed the LDAK software [78] to simulate several hundred genotype and phenotype datasets with three different heritability values: 0.05, 0.075 and 0.1. Consequently, we created 500 datasets consisting of 1000 SNPs, 2000 samples and a continuous phenotype controlled by a single SNP for each heritability value, respectively. Power was calculated as the proportion of datasets where the causal SNP obtained the highest MI value. To establish a proper value of *k* for the MI estimator, we systematically analyzed each dataset using *k*-values from 1 to 60. Despite Ross [52] and Kraskov et al. [79] both recommending a low value of k=3, our analyses indicate that such small values can be only considered for heritability values >0.1 (see Figure 4). Further, Figure 4 suggests that simulation datasets with smaller heritability values require a much higher *k*-value to successfully detect the causal SNPs of interest. By systematically analyzing different *k*-values, we established that a value of k=30 leads to the highest increase in power for the estimator based on the heritability values under study. We did not choose a higher value, since an increase in *k* results in a longer runtime for the estimator and may likewise cause problems if the sample size is not large enough.

### 3.2. Illustration of Background Associations and Its Correction Using APC

In information theory, mutual information (MI) is typically measured between two variables, $X^{1}$ and *Y*. Additionally, based on the chain rule of information [60], it is well known that the introduction of a new variable, $X^{2}$, might affect the relationship between $X^{1}$ and *Y*, thus increasing the MI between $X^{1}$ and *Y*. However, if the introduction of $X^{2}$ does not result in any new information, the corresponding MI value will not be affected [60].

**Figure 5:**
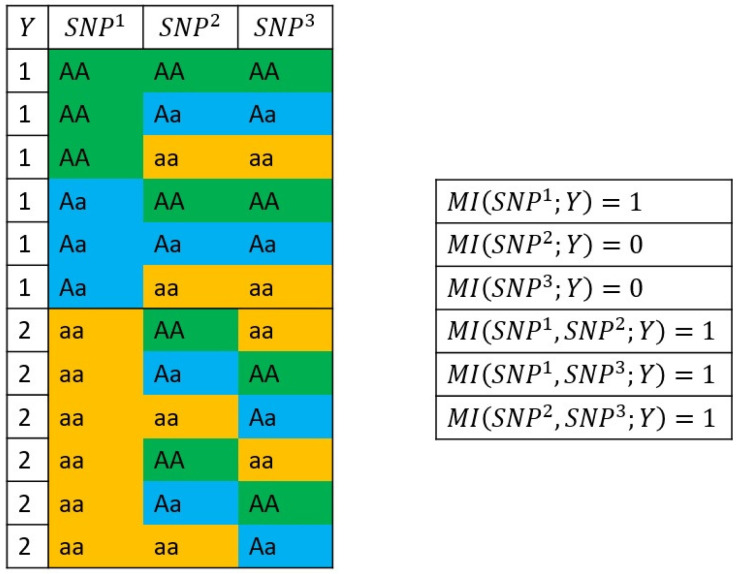
Example of MI values calculated from genotype data for three SNPs and twelve samples with a binary phenotype. The table cells are colored based on the genotype value of the SNP for the corresponding sample.

In case of SNP×phenotype associations, this property of the MI needs to be considered since only the introduction of an additional $SNP^{2}$ which increases the amount of information between $SNP^{1}$ and the phenotype *Y* should be taken into account for the detection of epistatic interactions. The reason for this is exemplified in Figure 5. It can be seen in Figure 5 that $SNP^{1}$ and *Y* have the maximum MI value of 1, indicating their perfect association. On the other hand, $SNP^{2}$ as well as $SNP^{3}$ have an association value of 0 to *Y*. Applying Equation (3) clearly shows that the introduction of $SNP^{2}$ or $SNP^{3}$ does not affect the amount of association between $SNP^{1}$ and *Y*, but on the other hand leads to a false interpretation of epistatic interactions. To deal with this problem, we apply the average product correction (APC) theorem [53], which ensures the elimination of negligible increments in the MI value of epistatic interactions measured using Equations (3) and (4).

Another important aspect of the usage of the MI metric in the context of epistatic interactions is its ability to detect the newly created relationship between a SNP pair and the phenotype, even though the single SNPs themselves might not show any association to the phenotype. This property of MI can be considered for measuring the level of association between $SNP^{2}-SNP^{3}$ and *Y* (see Figure 5).

**Figure 6:**
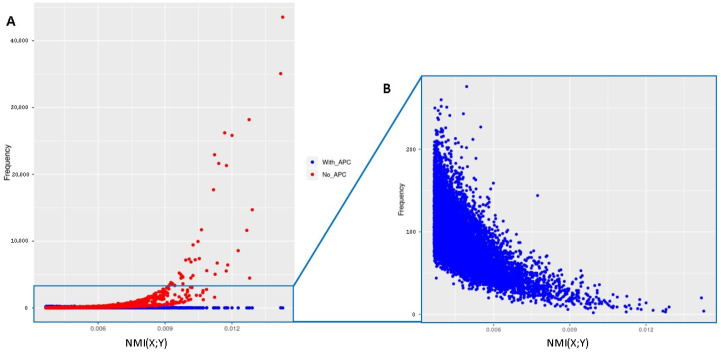
(**A**) shows the distribution of the SNP frequency and their association to the phenotype. The blue and red points stand for the frequency of the SNPs based on with and without the application of the APC, respectively. (**B**) only shows the frequencies for the interactions with APC.

To demonstrate the importance of the APC in the analysis of epistatic interactions, we further applied it for the correction of the MI values calculated using Equation (3) regarding the BT dataset. We considered the top million MI values indicating the epistatic interaction between the SNP pairs and the phenotype. Afterwards, for each SNP, we determined its frequency among the interactions. The frequency distribution of SNPs and their single association to the phenotype is shown in Figure 6A. As mentioned above, the frequency of several SNPs is over-represented, which arises from their single association to the phenotype. However, the application of APC dramatically reduces their frequencies in the epistatic interactions. This finding clearly suggests that, although these SNPs individually have a strong association to the phenotype, their epistatic interactions are negligible, as shown with blue points in Figure 6.

### 3.3. Bovine Tuberculosis Dataset

**Figure 7:**
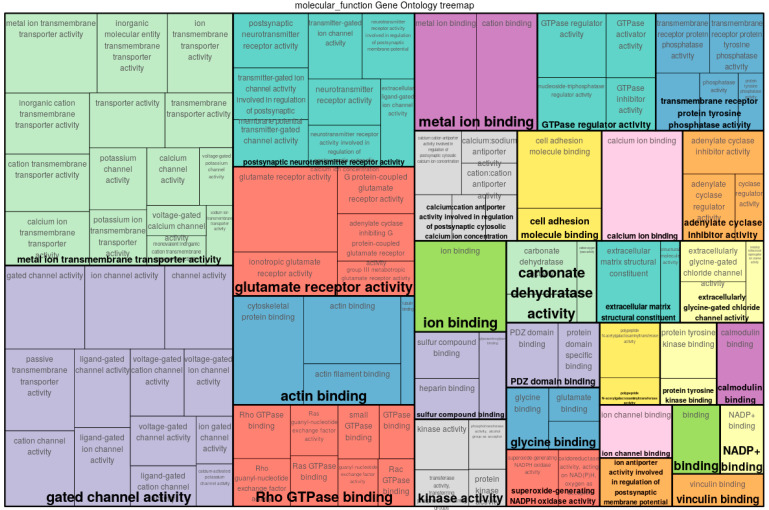
Gene Ontology (GO) treemap for genes associated with immunity to bovine tuberculosis. The boxes are grouped together based on the upper-hierarchy GO term, which is written in bold letters.

By applying MIDESP to the BT dataset, we first identified 10,774 single SNPs in total, with significant association to the phenotype. Taking all SNP pairs that contain at least one of those significant SNPs into account, for the epistatic interaction analysis, we identified 3,799,984 SNP pairs, which corresponds to 0.1% of all possible pairs under study. After that, we mapped these SNPs to their corresponding genes using the Ensembl database and a gene–gene interaction network was created, as suggested in [80]. Finally, according to this network, we detected 511 genes as MIDESP-significant and investigated their roles in bovine tuberculosis disease based on enriched Gene Ontology (GO) terms (see treemap depicted in Figure 7 and Supplementary Table S1).

The functional classification of these genes indicates that several of the GO categories represented in the treemap play essential roles in the immune responses towards bovine tuberculosis. Especially, metal ion transmembrane transporter activity and gated channel activity are the most significantly enriched terms, shown in the green and purple boxes in the treemap (Figure 7) obtained from the GO analysis, indicating the function of transmembrane proteins involved in the transportation of ions across membrane layers. Particularly, ion channel blockers are known for their therapeutic implications in drug-resistant mycobacterial infection, especially voltage gated calcium channels, which are important for the regulation of immunity against pathogens [81,82,83,84]. In this regard, increasing calcium influx by inhibiting the voltage gated channels in immune cells such as macrophages is highly associated with protective immunity, particularly in increasing the expression of genes involved in pro-inflammatory responses [84]. Other significant GO terms including actin binding, Rho GTPase binding, glutamate receptor activity and postsynaptic neurotransmitter receptor activity were also enriched in the treemap and their roles associated with *Mycobacterium tuberculosis* are described below. Firstly, actin filament, which is an important constituent of the cytoskeleton [85], is mainly associated with pro-inflammatory responses. A primary aspect of mycobacterial infection is the manipulation of actin filaments [86], notably inside the macrophages (immune cells engulfing the pathogens) of the host [87,88,89], thereby pointing out the importance of actin-binding protein regulation for enhancing the immune responses of the host. Several recent studies reported that neurotransmitters play essential roles in the activation or suppression of immune responses through the regulation of T-cell activity [90,91]. It is well known that T-cells play an important part in the defense of the host against mycobacterial infections [92,93,94]. Specifically, the neurotransmitter taurine was identified in relation with the susceptibility of cattle towards bovine tuberculosis [95]. Glutamate is likewise a neurotransmitter known for its effect on the immune system for the regulation of T-cell activity [96,97]. Finally, Ras homology GTPases (Rho GTPase) are proteins involved in the critical regulation of signaling pathways upon bacterial entry at the site of infection, and therefore are involved in innate immune responses, particularly in the multiplication of immune cells. It is essential to coordinate the immune responses at this point to prevent the neighboring tissue from taking damage from inflammation. Involved in the tight regulatory roles of multiple immune functions, these signaling proteins have been reported as targets of *Mycobacterium tuberculosis* during the host cell invasion, which might facilitate the pathogenesis of the bacteria [98,99,100].

### 3.4. Egg Weight Dataset

**Figure 8:**
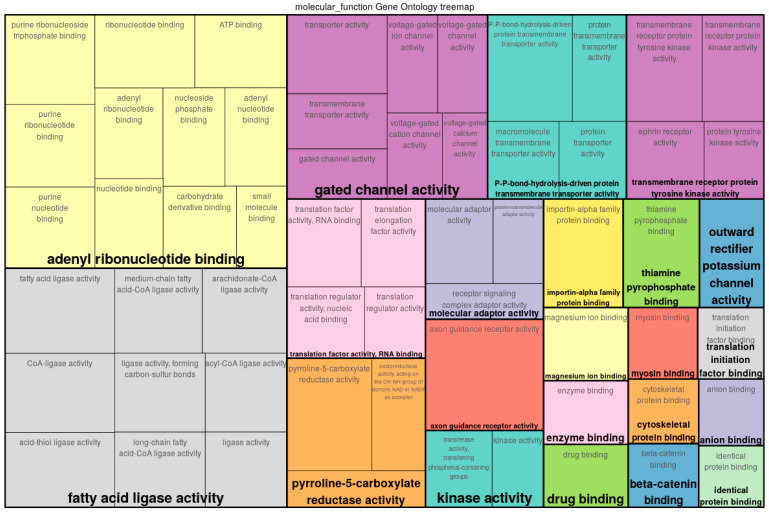
Gene Ontology (GO) treemap for genes associated with egg weight. The boxes are grouped together based on the upper-hierarchy GO term, which is written in bold letters.

Similarly to the previous dataset, MIDESP was used to analyze the EW dataset, which contains a quantitative phenotype. As a first step, we detected 3116 single SNPs that were significantly associated with the trait. Based on these SNPs, we measured the epistatic interactions between the SNP pairs and the phenotype and obtained 1,071,464 SNP pairs in total that equate to 0.25% of all possible pairs under study. After mapping these pairs to a gene–gene interaction network, we were able to identify 211 genes as MIDESP-significant. The analysis of their roles regarding egg weight was again carried out using their enriched GO terms (see treemap depicted in Figure 8 and Supplementary Table S2).

For egg weight, one of the major GO categories that emerged as a result of the gene set analysis was the fatty acid ligase activity. Fatty acid ligases belong to the ligase family of enzymes that take part in the biosynthesis of lipids [101]. Lipids constitute a major portion of the nutrients found in egg and are primarily contained in egg yolk, which constitutes 31% of the total egg weight [102]. Multiple genes encoding fatty acid ligases have been reported to play important roles in the laying performance of birds [103,104,105]. In this regard, we were able to discover many genes with molecular functions associated with acyl-CoA ligases, a group of enzymes, which are known to play important roles in the lipid synthesis by making the chemically inert fatty acids undergo activation into acyl-CoA [106]. This activation comprises an ATP-dependent reaction catalyzed by ligase enzymes in the presence of Mg${}^{2+}$ and CoA [107]. The usage of ATP and Mg${}^{2+}$ in this process can also explain the role of adenyl nucleotide binding and magnesium ion binding, two other categories identified in our analysis. Gated channel activity is another important GO term found in this analysis. These genes ensure the transportation of nutrients and minerals, which are required for the development of the egg. More importantly, for the synthesis of the eggshell, which contributes around 9% to the total egg weight [102], large amounts of calcium ions are supplied to the uterine fluid by transepithelial transport [102,108]. This transepithelial transport occurs with the help of ion channels, ion pumps and ion exchangers in the reproductive tract of birds and the energy required for these processes is provided by the metabolisms of ATP molecules [108]. Both nucleotide binding and gated channel activity have been reported in association with egg weight and eggshell development in chicken [54,55]. Furthermore, genes related to protein transmembrane transport activity were also identified in our analysis, which can regulate the transportation of the large number of proteins found in an egg [102,109]. The gene set analysis further reveals other activities pertaining to molecular bindings at different levels, which can play roles crucial for the development of egg.

### 3.5. Comparisons with Existing Methods

To investigate the performance of our new method, we were further interested in making pairwise comparisons between the results of our MIDESP, PLINK [57], GBOOST [110], epiGPU [111] and MatrixEpistasis [112]. Although all these methods take a genotype–phenotype dataset as input and report epistatic SNP pairs as result, their applicability differs based on the phenotypes. While MIDESP and PLINK can be applied to qualitative as well as quantitative phenotypes, the other methods are restricted to one type. GBOOST only deals with qualitative phenotypes, while epiGPU and MatrixEpistasis only analyze quantitative phenotypes. We chose these tools since they have previously been used for pairwise epistasis detection on real datasets, as well as for comparison studies [41,113,114,115,116,117,118,119], and ran them with their default parameters. It is important to note that for this comparison study, we applied MIDESP with and without APC correction. While the MIDESP without APC is in line with the conventional mutual information (MI)-based methods for epistasis detection [39,46,80,120], the incorporation of the APC approach is completely novel and necessary to separate the correct epistatic signals from the background.

The results of this comparison are twofold. First, we compared the results of our method using the BT dataset with those of PLINK, GBOOST and the conventional MI-based metric, since the existing MI-based approaches are only applicable to qualitative phenotypes [39,46,80,120]. Second, we compared the predictions of MIDESP on the quantitative EW dataset with those of PLINK, epiGPU and MatrixEpistasis. However, our attempt to apply MatrixEpistasis to this dataset was not successful due to its very high memory consumption (700 GB of memory was not enough).

The application of these methods results in the detection of strongly varying numbers of SNP pairs as epistatic interactions, which are given in Table 2.

**Figure 9:**
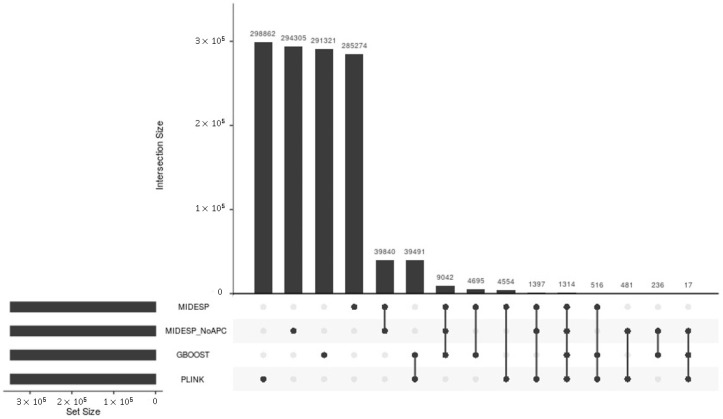
Number of epistatic SNP pairs detected for the BT dataset and their overlap between four methods represented in matrix layouts using the UpSet technique [121]. Black circles in the matrix layout indicate which methods are part of the intersection.

**Figure 10:**
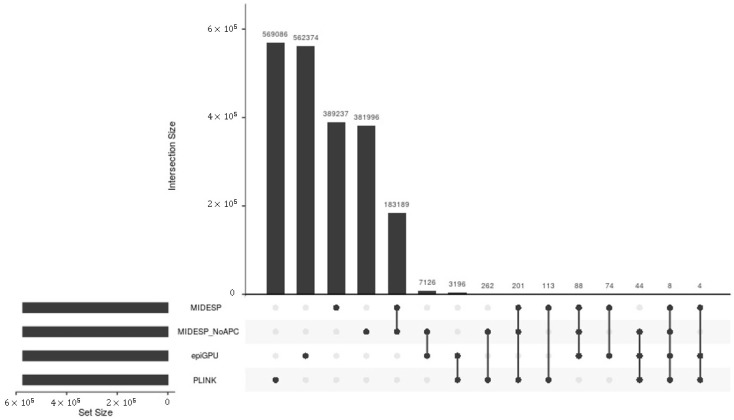
Number of epistatic SNP pairs detected for the EW dataset and their overlap between four methods represented in matrix layouts using the UpSet technique [121]. Black circles in the matrix layout indicate which methods are part of the intersection.

To make the predictions of the methods comparable, in this comparison analysis for both types of the traits, we considered 346,632 and 572,914 epistatic SNP pairs, which corresponds to the minimum numbers of SNP pairs found by GBOOST and epiGPU for the BT and EW datasets, respectively (see Table 2). Based on these top SNP pairs, we further performed an overlap comparison between the methods and visualized the results using UpSet plots in Figure 9 and Figure 10, respectively. Although all of these methods perform a search for epistatic SNP pairs, Figure 9 and Figure 10 clearly show that they provide quite distinct results, with only little overlap between them. This finding is in line with the comparison study performed in [113], which also reported divergent results between different methods for epistasis detection. The reason for that may be explained due to differences in the underlying algorithms, even though the three other methods are ultimately based on logistic and linear regression, respectively. While PLINK performs a regression with an interaction term and tests whether the coefficient for the interaction is significant, GBOOST considers the difference in the likelihood of a linear model with interaction compared to that of a model without as a sign for epistasis using approximations to speed up the process and filter out SNP pairs. On the other hand, epiGPU treats the different genotype combinations as different classes and calculates differences in the class means compared to the population mean.

Consequently, the results of this overlap analysis clearly demonstrate that these methods carry quite distinct information about epistatic interactions, due to the different measures they use. The finding of this comparison analysis is also in agreement with the previous study [113] and indicates that each of these methods takes into account a different manner of epistatic interactions, and thus they can work complementarily with each other.

## 4. Discussion

It has previously been shown that information theoretical methods based on mutual information (MI) are powerful approaches for the detection of epistatic interactions [39,41,43,44,45,46]. Not only here, but also in many other fields, mutual information has been used as an effective measure for the association between variables including linear as well as non-linear relationships [53,61,63,69,122,123,124,125]. However, the general applicability of a method, particularly in the field of animal and plant breeding, requires it to be usable for qualitative as well as quantitative phenotypes. For this reason, an extension of the previous MI methods, which are only suitable for qualitative traits, is required, and thus we adapted the estimator developed by Ross [52] for the case of MI between discrete and continuous variables. As shown in Section 3.1, the estimator can be successfully used to detect associations between SNPs and quantitative phenotypes. Surprisingly, we found that a higher *k* value improves the power of the measure when it comes to the detection of associations involving traits of a low heritability (see Figure 4), although previous studies recommended a small value of *k* for this purpose [52,79].

The progress over the last decade in the field of genome sequencing and genotyping arrays has increased the amount of available genotype data tremendously. With the ever-increasing amount of data, however, comes the challenge to provide tools that can handle such datasets in a feasible computation time. To address this challenge, redundant SNPs can be removed through LD pruning with a high threshold [56] (see Section 2.1) but there are still very high numbers of SNPs in a dataset to analyze all possible pairs. A commonly used approach to reduce the computational effort is to preselect sets of SNPs that are deemed as important and only analyze those, as is performed by BOOST and other methods [38,126,127]. Such an approach can potentially eliminate some SNPs which nevertheless influence the phenotype in interaction with another SNP. To overcome this problem, in our proposed method, we consider all SNP pairs where at least one SNP shows a strong association signal to the phenotype, which ensures a tractable computational time for MIDESP. For this step, we followed the approach outlined by Gültas et al. [63,64] to separate the SNPs with strong association signals from the remaining SNPs (see Section 2.2.3).

However, the sole consideration of SNPs with strong association signals could lead to a wrong interpretation in epistasis analysis since the NMI values are influenced by the association of the single SNPs with the phenotype, as we demonstrated by means of an example in Figure 5. This can result in the detection of false positive interactions that are only found due to the effect of one SNP. To minimize this influence, the application of the average product correction (APC) is essential, which was developed by Dunn et al. [53]. Moreover, Meckbach et al. [69] showed that the APC is universally applicable to MI-based methods to estimate the expected (background) association level of a variable. Although the concept of the APC theorem seems to be suitable for our purposes, its application would require a huge additional computational overhead. Therefore, we followed a strategy based on the three different distributions of the SNPs (see Section 2.2.3) for the efficient estimation of the expected level of background associations of SNPs. In particular, in Equation (9) we randomly choose the SNP $X^{l}$ from the set of SNPs that follow the $G_{2}$ distribution. This process ensures that the expected background level of SNP $X^{i}$ is clearly higher than it would be if estimated based on the whole set of SNPs. Consequently, the removal of the estimated background associations (APC values) from the obtained NMI values results in the separation of correct epistatic signals caused by SNP pair and phenotype interactions from background signals. Being of particular interest, in our analysis, we illustrated the effectiveness of the APC based on the BT dataset in Figure 6. This analysis reveals that the over-representation of SNPs with a large single effect among the pairs with the highest NMI values can be considerably reduced based on the application of APC, which in turn results in the detection of further associated genes.

The results we present in this study for the two different genotype–phenotype datasets show that the functional analysis of the detected genes provides essential information to decipher the genetic background of the traits under consideration. Surprisingly, we were able to clearly identify higher numbers of associated genes for the bovine tuberculosis dataset with a qualitative trait than for the egg weight dataset with a quantitative trait. The reason for this can be explained due to the large difference in the initial numbers of SNPs in both datasets (see Table 1). In comparison to the large numbers of associated genes detected by MIDESP, both original studies [58,59], in which the datasets were published, were only able to find two significantly associated genes for the respective dataset using standard GWAS approaches.

To further investigate the impact of the APC theorem in the epistasis analysis and to gain more insight into its influence on the detection of genes, we analyzed both datasets with and without the application of the APC (see Figure 6). It can be assumed that without the APC, the results of MIDESP are in line with previous methods that utilized MI for the detection of epistatic interactions for qualitative phenotypes [39,46,80,120]. The analysis reveals that the application of the APC leads to a considerable increase in the number of associated genes for both datasets. For example, only 135 and 177 significant genes were found for the BT and EW datasets without using the APC, respectively. However, the correction of the background association using the APC results in the detection of 511 and 211 associated genes, respectively. The comparison of these genes showed that while 59 genes overlap for the BT dataset, 51 overlapping genes are found to be significant for the EW dataset. The functional analysis of these genes based on their GO categories reveals that many of the identified genes are involved in the regulation of the immune system regarding bovine tuberculosis, with several of the functions having a reported association with mycobacterial infections. The genes that were detected for the egg weight dataset, on the other hand, are mainly related to the production of important components of the egg and the transportation of these components to the uterine fluid. Overall, our results indicate that MIDESP is an effective method for the detection of epistatic interactions that for the first time enables the analysis of quantitative phenotypes using MI and further extends the existing information theoretical methods by correcting the influence of background associations of the SNPs through the application of the APC theorem.

## 5. Conclusions

Today, it is well established that MI-based methods are suitable and effective approaches for the detection of epistatic interactions for qualitative phenotypes. However, these approaches are not directly applicable for quantitative phenotypes, although epistatic interactions for quantitative traits are of great interest in life sciences. To address this limitation of the existing MI-based methods, we extend their applicability for the first time in this regard to quantitative phenotypes using a *k*th-nearest neighbor-based estimation technique. Another important challenge for the detection of epistatic interactions is the control of the effect of background associations in the genotype–phenotype datasets, which lead to false interpretation and thus the overestimation of the role of some SNPs in the epistasis. To deal with this issue, in our proposed method, MIDESP, we additionally modeled these background associations by adopting the APC theorem, which we extended for the multivariate mutual information. Our findings show that the MIDESP algorithm is applicable to genotype–phenotype datasets with qualitative as well as quantitative phenotypes in a tractable computational time. For example, the analysis of the BT dataset took only 36 minutes, while the analysis of the EW dataset was completed in 105 minutes. These runtimes were achieved on a dual Intel^®^ Xeon^®^ Gold 6138 Processor using 70 threads. Our results further indicate that the biological processes of the identified genes in the BT and EW datasets are strongly related to both bovine tuberculosis and the egg weight of chickens, respectively. To the best of our knowledge, MIDESP is the first method that models epistatic interactions using the MI metric for both qualitative and quantitative phenotypes and explicitly corrects for background associations. The program is written in Java and is freely available as a JAR file from https://github.com/FelixHeinrich/MIDESP, accessed on 14 September 2021.

## Figures and Tables

**Table 1:** Overview of the datasets used in our study.

Dataset	Phenotype	#Samples	#SNPs	#SNPs after Filtering	#SNPs after LD Pruning
Bovine Tuberculosis	Qualitative	1151	617,885	616,398	358,086
Egg weight	Quantitative	1063	580,961	294,705	139,101

**Table 2:** Number of SNP pairs that were found to be an epistatic interaction by the different methods. BT and EW stand for bovine tuberculosis and egg weight, respectively.

Dataset	#MIDESP	#MIDESP_NoAPC	#PLINK	#GBOOST	#epiGPU
BT	3,799,984	3,799,984	4,982,695	346,632	-
EW	1,071,463	1,071,463	1,817,817	-	572,914

## Data Availability

The datasets that we analyzed as well as the source code for MIDESP can found in the repository https://github.com/FelixHeinrich/MIDESP, accessed on 14 September 2021.

## Supplementary Materials

The following are available online at https://www.mdpi.com/article/10.3390/biology10090921/s1, Table S1: results of the gene set analysis for the BT dataset, Table S2: results of the gene set analysis for the EW dataset.

## Abbreviations

The following abbreviations are used in this manuscript:

SNP	Single-nucleotide polymorphism
GWAS	Genome-wide association studies
MI	Mutual information
NMI	Normalized mutual information
BT	Bovine tuberculosis
EW	Egg weight
APC	Average product correction
FDR	False discovery rate
GO	Gene Ontology
